# Effects of Pesticides on the Survival of Shredder *Nectopsyche* sp. (Trichoptera) and Leaf Decomposition Rates in Tropical Andes: A Microcosm Approach

**DOI:** 10.3390/toxics10120720

**Published:** 2022-11-24

**Authors:** Christian Villamarín, Miguel Cañedo-Argüelles, Constanza Carvajal-Rebolledo, Blanca Ríos-Touma

**Affiliations:** 1 Grupo de Investigación Biodiversidad, Medio Ambiente y Salud (BIOMAS), Universidad de Las Américas, Quito 170503, Ecuador; 2 Ingeniería Ambiental, Facultad de Ingenierías y Ciencias Aplicadas, Universidad de Las Américas, Quito 170503, Ecuador; 3 FEHM-Lab, Institute of Environmental Assessment and Water Research (IDAEA-CSIC), 08016 Barcelona, Spain

**Keywords:** environmental risk, aquatic macroinvertebrates, highland Andean rivers, pesticides, alnus, Engeo, Chlorpyrifos

## Abstract

Andean streams are becoming increasingly impacted by agricultural activities. However, the potential effects of pesticides on their aquatic biodiversity remain unassessed. In order to address this knowledge gap, we conducted an experiment over 37 days in microcosms to assess the effect of two pesticides commonly used in Ecuador (Engeo and Chlorpyrifos) on the aquatic insect *Nectopsyche* sp. (Trichoptera: Leptoceridae) at 0, 0.10, 5 and 10 μg L^−1^ concentrations. The highest concentration corresponds to the maximum concentration allowed by the Equatorian legislation. We assessed insect mortality every 24 h, with leaf litter decomposition rates of organic matter determined by deploying Andean alder *(Alnus acuminata*) dry leaf packs in the microcosms. We found significant mortality of *Nectopsyche* sp. at high concentrations of Chlorpyrifos, whereas leaf litter was not significantly affected by any of the treatments. We conclude that the environmental legislation of Ecuador might not be fully protecting aquatic biodiversity from pesticide pollution. Further studies are needed, especially when considering that the maximum permitted concentration is very likely exceeded in many areas of the country. We also suggest that the maximum permissible values should be reviewed, considering each pesticide individually.

## 1. Introduction

Population growth exerts a high pressure on ecosystems, not only in terms of water and land use demand, but also in terms of agricultural production [1]. In order to maintain a high crop productivity, the farmers are obliged to use pesticides to manage pests [2], but in many cases, the overuse of pesticides causes environmental or health problems [3,4,5,6].

Pesticides are generally stable, organic, toxic compounds which are used to control target organisms that can affect crops. However, of the amount of pesticide applied in a cultivated field, only a small part reaches its target; the rest is dispersed into the environment [7]. This allows these compounds to move through environmental compartments such as soil, air, water, groundwater or biota [4]. Furthermore, some pesticides can remain in environmental compartments for long periods [8] and bioaccumulate in living organisms [4,9].

There are several ways that pesticides disperse during application. Some examples include runoff, leaching, spray drift, and droplet spray drift [5,7], all of which can lead to health and environmental risks. At an individual or population level, this risk is understood as an adverse health effect, resulting in decreased growth and reproduction rates, mobility reduction and, potentially, the exposed organism’s death [10,11]. These effects have been identified in a variety of organisms, from soil invertebrates to birds, fish and mammals [4]. Therefore, pesticides have high bioaccumulation and transfer rates in ecosystems [12].

Although pesticides are not directly applied to aquatic ecosystems, there is evidence of pesticide presence in mollusks, amphibians and fish [9,13,14]. Pesticides enter aquatic ecosystems mainly via runoff, lixiviate and spray drift from nearby crops [14,15]. Once they enter the aquatic environment, the persistence of these compounds contributes to the transference of pesticides by ingestion of contaminated dietary sources in the trophic chain [13,16]. This can not only negatively affect biodiversity, but also impact the functionality of the ecosystem [17]. For example, pesticides can lead to a loss of invertebrate shredders, thereby reducing leaf litter decomposition in streams [18,19]. This is important because organic matter decomposition is essential to the biogeochemical cycles of ecosystems, as well as to maintaining the ecosystem’s health and functionality.

Andean rivers are born in the upper basins (around 5000 m asl) and flow through the land for many kilometers, crossing cities, crops and farms. These rivers provide water to the lower basin, which is essential to Andean ecosystems and human populations. The population in the Andean basins is increasing, causing changes in the land use in order to supply food and water to the cities [20,21,22,23]. Thus, the use of agrochemicals is intense in agricultural areas, degrading the water quality in these regions [3,24]. This increasing use of pesticides, combined with a poor technical application, could significantly affect the environment. For example, residual agrochemical tanks washed in rivers and excessive fumigation concentrations are frequent in the agricultural lands of the Andes [24]. Additionally, it is common to find used pesticide containers in rivers [23]. However, the potential effects of agronomical practices on the functionality of the surrounding aquatic ecosystems remain unassessed in the Andes.

In Ecuador, the use of pesticides has greatly increased due to growing agricultural activity [6,15], with Engeo and Chlorpyrifos being widely used. Engeo is composed of thiamethoxam and lambdacialothrin, which are pyrethoid substances that alter the nervous system and present a high residuality in insects [25]. On the other hand, Chlorpyrifos is a synthetic organophosphate acetylcholinesterase inhibitor. It is one of the most widely used pesticides in agriculture and is considered a moderately toxic substance, which has chronic affections on terrestrial and aquatic insects [25]. Studies have shown that these substances are poorly soluble in water, meaning that they tend to volatilize and, on other occasions, concentrate in river sediments, affecting aquatic invertebrates that feed on them [26]. In the event that Engeo and Chlorpyrifos do not volatilize, their permanence in water can span from days to weeks [27].

Even though there are protective water policies that include maximum allowable pesticide concentrations in rivers to protect the biodiversity, these values has been adapted from other countries’ policies due to the absence of local studies [28]. Having information on the potential effects of pesticides on the aquatic biodiversity of Andean rivers is urgent, since they are biodiversity hotspots subjected to an increasing human pressure. Although there are several studies on the presence or dispersion models of pesticides in Ecuador [6,24,29], the effects of pesticides on aquatic macroinvertebrates remain unassessed. The aim of this study was to evaluate the effects of pesticides on the leaf litter decomposition rates and the mortality of *Nectopsyche* sp., a shredder/collector Trichoptera commonly found in the Andean rivers [30]. We hypothesized that Engeo and Chlorpyrifos (two commonly used pesticides) would have lethal effects on *Nectopsyche* sp. at the maximum permitted concentrations by the Ecuadorian environmental legislation, and that their use would lead to a decrease in the decomposition rates of the leaves of Andean Aliso (*Alnus acuminata*), which is a common tree in the riverine forest in the Andes that has been used as a model for aquatic decomposition in Andean streams [31].

## 2. Materials and Methods

### 2.1. Microcosm Construction

**Figure 1:**
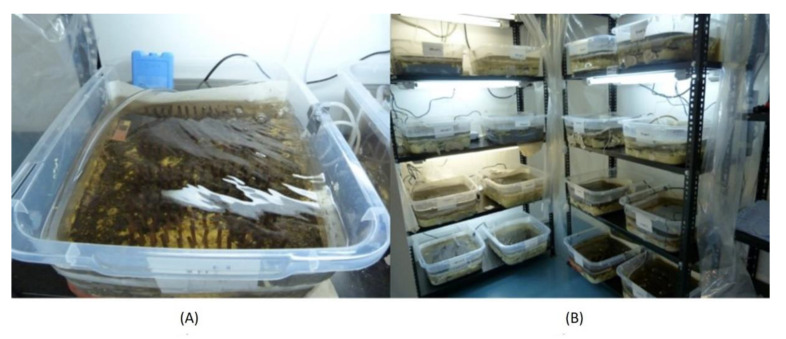
(**A**) Microcosm design. (**B**) Distribution of the microcosm in the experimental room under controlled light and temperature conditions.

We recreated the river habitat (Figure 1A) in plastic trays (47 × 26 × 37 cm). Each microcosm had two pumps, one air pump to maintain an adequate oxygen level, and another to recirculate the water. To recreate the river habitat, sand and gravel were sampled from the sampling site and transferred into the tray. Before the experiment began, the sand and gravel were washed with water and dried at room temperature. To simulate day and night light conditions in the equator, timers were programmed with 12 h of light and 12 of darkness. Room temperature was maintained between 18 +/−1 °C.

### 2.2. Nectopsyche sp. Collection

The *Nectopsyche* sp. individuals were collected in the Alambi river, which is a tributary to the Guayllabamba river. The sample site is located at 2830 m asl, and is subjected to very low human impact. We took samples using a D net and kicking in different habitats (sand, litter matter and rocks). Samples were placed on white trays. The individuals were separated and carefully removed until 240 individuals were obtained with tweezers. Later, the individuals were transported to the laboratory in containers with river water, litter matter and cooler packs in order to maintain the temperature of the river. Then, they were left in the river water for a 12 h acclimatization period in an air-conditioned room at 18 °C, before our microcosm experiments began in the laboratory facilities (Figure 1B). After inspecting some adults grown in our mesocosm facility, we confirmed that the individuals used in this experiment most likely belong to a new species that has not yet been described (Ralph Holzenthal, personal communication).

### 2.3. Leaf Packs Construction

Recently fallen (24 h) Andean Aliso (*Alnus acuminata*) leaves were collected from the floor in the Metropolitan Park of Quito. The litter matter was placed in cardboard boxes and dried at room temperature for approximately two weeks. Leaf packs made up of a nylon net of 0.5 cm, with a mesh size of 10 × 7 cm, were used to analyze the leaf decomposition rates in the microcosms. The ends of the leaf packs were heat-sealed, each of them containing 5 g of dried leaves. Each microcosm contained 6 leaf packs, which were placed in the microcosms four days prior to the start of the experiment.

### 2.4. Experimental Design

The experiment was conducted in an air-conditioned room (18 °C) with a 12:12 photoperiod. Two pesticides were used in the experiments (Engeo and Chlorpyrifos), with three treatments and three controls.

The lowest treatment had a pesticide concentration of 0.1 Μg L^−1^, which can easily be found in rivers due to agricultural runoff [17,25]. The next treatment had a concentration of 5 μg L^−1^; it was selected as a middle value between the low and high treatments. Finally, the high treatment had a concentration of 10 μg L^−1^, which corresponds to the environmental legislation of Ecuador TULSMA [28] maximum allowable values, to preserve flora and fauna. In order to assess changes in the leaf decomposition rate of *Nectopsyche* sp., two different experimental setups were used for each treatment: one only with leaf packs, and the other with *Nectopsyche* sp. individuals and leaf packs. Each treatment had three replicates.

### 2.5. Variables Measurements

During the experiment, the physico-chemical variables were measured every 24 h. Conductivity and pH were measured using a Ysi Pro10 multiparametric probe. Dissolved oxygen and temperature were measured with a Ysi ODO probe (Yellow Springs, OH, USA).

One leaf pack was removed from the microcosms every week. In order to obtain the dry weight, the leaf content was removed from the pack and placed in a stove at 75 °C for 24 h. Then, the content was placed in a muffle at 550 °C for 4 h in order to obtain the ash-free dry mass. The weight of the organic matter was measured with an analytical balance. The decomposition velocity coefficient of each leaf pack was then calculated (Morgana and Prato, 1994).

The number of individuals were counted every 24 h during the experiment. The dead *Nectopsyche* sp. individuals were removed and registered.

### 2.6. Data Analysis

We analyzed the global differences in the survival of *Nectopsyche* sp. and leaf decomposition between treatments using ANOVA and Tukey tests, after checking that the assumptions of normality and homoscedasticity were met. To assess changes in survival and leaf decomposition over time in the different experiments, we followed the statistical framework developed by Feld, Segurado and Gutiérrez-Cánovas [32]. First, we built Random Forests, using survival as the response variable, and treatment intensity as well as all of the measured environmental variables as predictors. To do this, we used the function “rfsrc” from the R package MuMIn [33]. Then, we built generalized linear models using survival and leaf decomposition as the response variables, with all of the variables (and their interactions) selected as important by the Random Forests, as predictors. Finally, we ran all possible models and ranked them by their AIC using the “dredge” function in the same package.

## 3. Results

### 3.1. Survival

**Figure A1:**
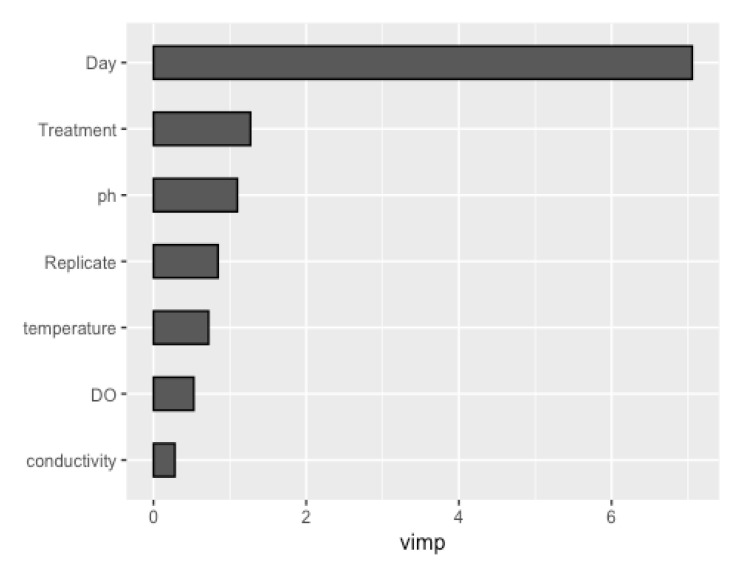
Variable importance explaining changes in mortality according to Random Forests.

**Figure A2:**
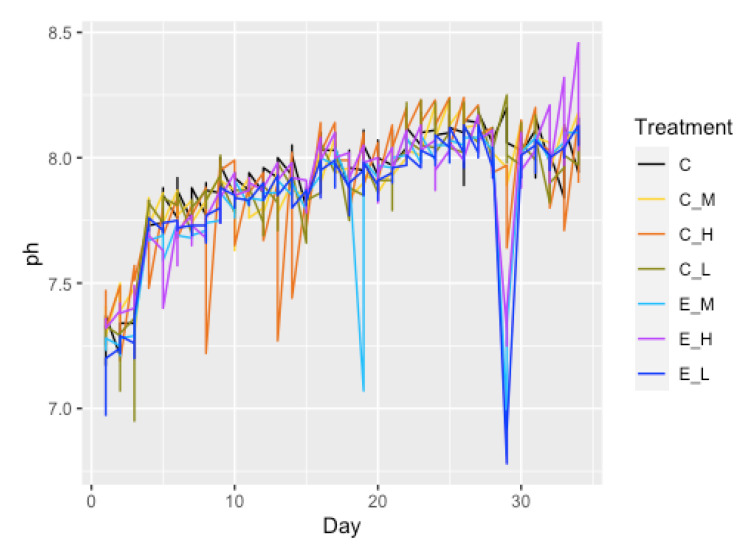
Variation in pH along time between treatments.

According to Random Forest (Appendix A, Figure A1), time was the most important variable explaining differences in mortality (variable importance = 7.05), followed by treatment (variable importance = 1.27) and pH (variable importance = 1.09). The best model (R^2^ = 0.69, weight = 0.47) included all three variables and their interactions. According to the model coefficients, there was a significant relationship between mortality and the treatments, as well as their interaction with pH, for all three doses of Chlorpyrifos and for the high dose of Engeo. The interaction with pH was due to a logarithmic increase in pH in all treatments over time (Appendix B, Figure A2).

**Figure 2:**
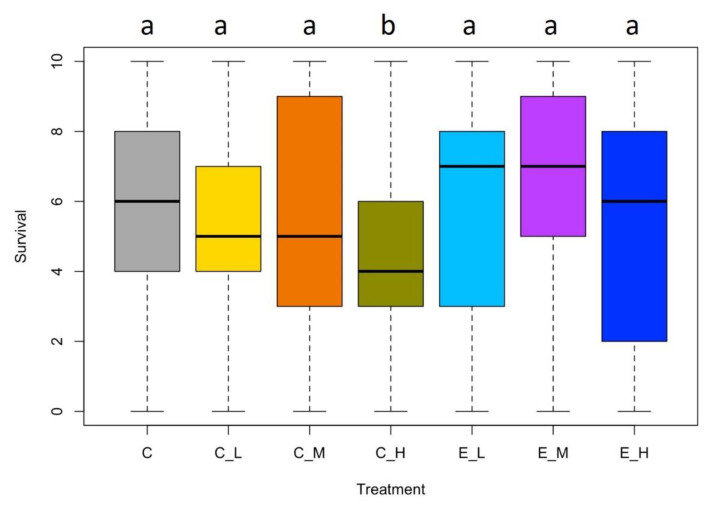
Survival analysis of *Nectopsyche* sp. related by different concentrations of Chlorpyrifos (C_ L: Low, C_M: Middle; C_H: High) and Engeo (E_L: Low, E_M: Middle; E_H: High). C corresponds to the experimental control. The letters on the box plots correspond to significant differences in the Tukey test between treatments and the control, no shared letters show significant differences.

According to the ANOVA test, there were significant and strong differences between treatments (F-value = 7.11, *p*-value = 2.26 × 10^−7^). However, the Tukey post-hoc test revealed that only the high dose of Chlorpyrifos resulted in a significantly lower survival when compared with the control treatment (diff = −1.39, *p*-value = 0.01; Figure 2). There were also significant differences between the Chlorpyrifos and the Engeo treatments, with the former leading to lower survival rates (Figure 2).

**Figure 3:**
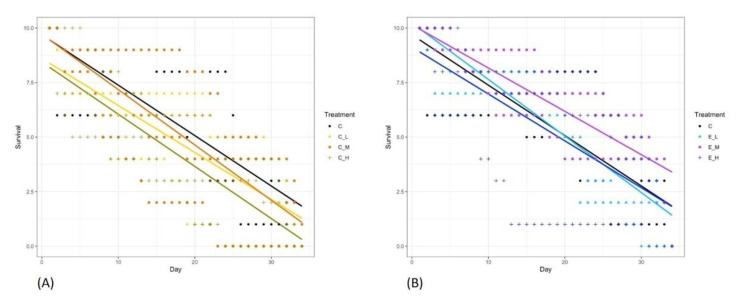
Survival analysis of *Nectopsyche sp.* related by experiment time with Chlorpyrifos (**A**) and Engeo (**B**). C corresponds to experimental control, C_ corresponds to Chlorpyrifos low (L), middle (M) and high (H) concentrations and E_ corresponds to Engeo low (L) middle (M) and high (H) concentrations.

In the case of the Chlorpyrifos treatment (Figure 3A), survival declined significantly with time in all treatments: low (R^2^ = 0.77, *p*-value < 2.2 × 10^−16^), moderate (R^2^ = 0.73, *p*-value < 2.2 × 10^−16^) and high (R^2^ = 0.44, *p*-value = 1.14e-14). In the case of the Engeo treatment (Figure 3B), survival also declined significantly with time in all treatments: low (R^2^ = 0.60, *p*-value < 2.2 × 10^−16^), moderate (R^2^ = 0.60, *p*-value < 2.2 × 10^−16^) and high (R^2^ = 0.67, *p*-value < 2.2 × 10^−16^). Finally, survival declined significantly with time in the control treatment (R^2^ = 0.68, *p*-value < 2.2 × 10^−16^).

### 3.2. Leaf Decomposition

**Figure A3:**
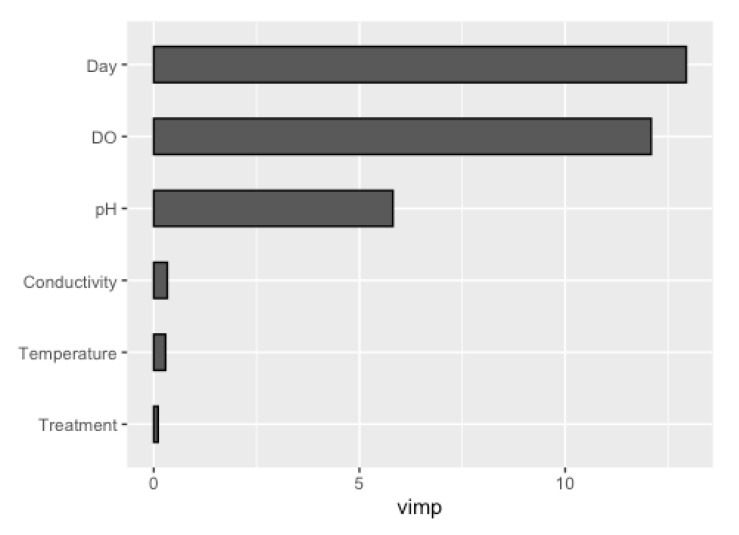
Variable importance explaining changes in leaf decomposition according to Random Forests.

According to Random Forest (Appendix C, Figure A3, time was the most important variable explaining differences in leaf decomposition (variable importance = 12.94), followed by dissolved oxygen (variable importance = 12.09), and then pH (variable importance = 5.81). The best model (R^2^ = 0.60, weight = 0.59) included all three variables and their interactions. According to the model coefficients, there was a significant relationship between leaf decomposition and time, as well as an interaction of time with pH and dissolved oxygen.

**Figure 4:**
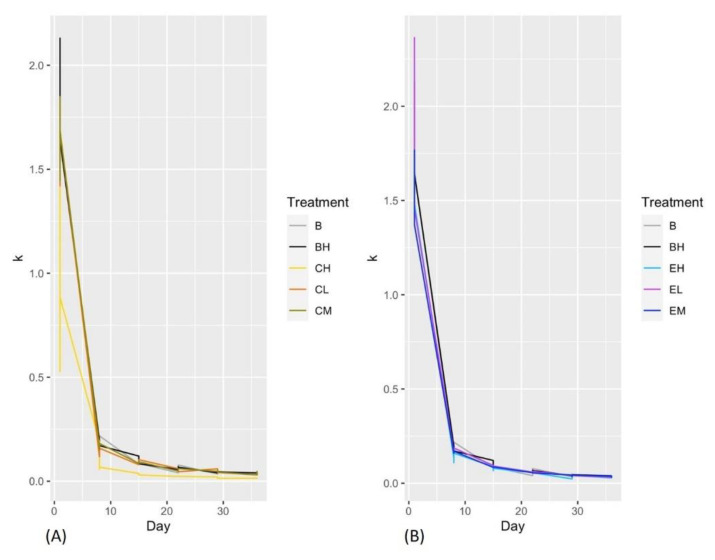
Decomposition rate in experiments with Chlorpyrifos (**A**) and Engeo (**B**). The letter Bin the figure legend corresponds to the experimental control without *Nectopsyche* sp. and BH corresponds to experimental control with *Nectopsyche* sp. C corresponds to Chlorpyrifos low (CL), middle (CM), and high (CH) concentrations. E corresponds to Engeo low (EL), middle (EM), and high (EH) concentrations.

The ANOVA and Tukey tests showed no significant differences in leaf decomposition between treatments. There was a significant decrease in the leaf decomposition rate over time for all treatments, including the control (Appendix C, Figure A3, with a very drastic reduction during the first 8 days of the experiment (Figure 4).

## 4. Discussion

Our results show that *Nectopsyche* sp. individuals were significantly affected by the addition of pesticides, especially at high concentrations of Chlorpyrifos, and that their response was time-dependent. The decomposition rates of organic matter were not significantly affected by treatment, but showed a significant response to differences in pH between treatments. Although we did not detect strong effects of the pesticides on leaf litter decomposition, the increase in *Nectopsyche* sp. mortality suggests that chronic pesticide pollution could eventually affect it.

The environmental targets in the Latin American region are focused on biodiversity, habitat degradation, pollution, climate change and human welfare [34]. However, the legislation in Andean countries is lax, with scarce controls to achieve adequate environmental health. For example, the environmental control entities do not have any regular monitoring or quality assessment of water bodies which would aid in understanding the effect of agriculture on Andean streams [35]. The existing information on water quality comes from grey literature, environmental consultancies or research [35,36]. Therefore, the effect of anthropogenic activities on riverine biodiversity and ecosystem functioning could easily be underestimated.

Agrochemicals used on crops are transported to aquatic ecosystems through different pathways, and affect non-target organisms, such as some aquatic insects [33,37]. For example, in alignment with our study, Morabowen et al. (2019) found that *Nectopsyche* decreased along an agricultural land use gradient. It is important to notice that some individuals survived our experimental treatments, even at the highest pesticide concentration. This means that pesticides have the potential to bioaccumulate in *Nectopsyche* and to be transferred into the river food chain [4,38]. Concordantly, previous studies have demonstrated that long term pesticide effects can be detected in freshwater ecosystems [39,40]. Taking into account that Chlorpyrifos is especially toxic to aquatic life [7,12,24], with some studies showing long term effects on aquatic biodiversity [41], the potential bioaccumulation of Chlorpyrifos in the environment through aquatic insects deserves to be further studied.

In our study, Chlorpyrifos caused a significant mortality of the caddisfly *Nectopsyche* sp. at the highest concentration used (10 μ gL^−1^), as we hypothesized. However, both Chlorpyrifos and Engeo are classified with the same toxicity in the national legislation [28], and we clearly observed differences in the effects of both on *Nectopsyche* sp. Thus, it is important to develop locally adapted maximum permissible values for the main pesticides used in Ecuador, in order to adequately protect water quality and conserve aquatic biodiversity.

Considering that *Nectopsyche* is very sensitive to pollution, our results suggest that current legislation could be protecting aquatic biodiversity to some extent. However, at the ecosystem level, it is expected that more sensitive taxa will be affected. Current regulations indicate that a maximum concentration of 10 μ gL^−1^ for total organophosphates is allowed in water. However, considering that bad practices are common [24,40], this may not be enough to adequately protect water quality [6]. In Latin America (including Ecuador), pesticides are used in agriculture with almost no technical expertise, leading to excessive pesticide application, which causes the pollution of soils, water and sediments [6,24,42]. Additionally, pesticides such as Engeo and Chlorpyrifos are easily accessible due to the lack of regulation and their low cost. Therefore, the maximum permissible pesticide concentrations may often be exceeded [6,15,16]. In addition, there is no information on the concentration of organophosphates in the water, which can affect biodiversity and ecosystem functioning [4,43]. Finally, the accumulation of pesticides in the sediment is not considered in the environmental legislation. This is an important point, because sediment accumulation can affect the biotic and abiotic characteristics in the short and long term [16,39], as well as the legacy effects of pesticides on freshwater ecosystems [41,44]. The lack of legislation regarding pollutants in sediments is an important issue to be addressed in order to effectively protect freshwater ecosystems in Ecuador.

According to our data, there were no differences in leaf litter decomposition between treatments, but decomposition rates changed with time. For the first eight days, the decomposition rate was high. This could be related to the shredder’s activity and the availability of food resources in the microcosms. However, we did not measure the effect of pesticides on microbial communities, which can be largely responsible for leaf litter decomposition [31,45]. Since pH can significantly affect the microbial decomposition of organic matter in rivers [46,47], the pH increase throughout the experiment could partly explain the low decomposition rates observed after the eighth day. Nonetheless, the microbial activity in rivers is one of the most important factors in decomposition rates, and is especially influenced by land use changes [46]. However, the agrochemicals input by runoff cause the eutrophication process or water chemical changes, the microbial activity is altered and the decomposition rate could be decreased [31,48]. It is important to denote other factors that intervene in the components of decomposition. The forest quality is highlighted as a factor that determines the importance of decomposers by the K_g_/K_f_ quotient, and in Andean rivers with high forest quality, the shredders are determined to be important to the decomposition rates [48], while the importance of microorganisms increases in impacted rivers [49,50]. This is one of the limitations of our study. We encourage future researchers to study changes in the composition and biomass of microbial communities, as well as how they might affect organic matter processing in Andean streams affected by pesticide pollution. Another limitation of our study is that we focused on a single species, neglecting potential trophic interactions. In this regard, future studies combining mesocosm experiments with field studies could be helpful in assessing how pesticide pollution might affect species interactions and the biological routes of pesticides in riparian and aquatic compartments [51].

Our study includes the genus *Nectopsyche* (Trichoptera: Leptoceridae) as a sentinel organism. According to previous studies in highland Andean rivers, *Nectopsyche* is among the organisms which are most sensitive to environmental changes [52,53]. Concordantly, our study proves that it this a good model organism for use in laboratory toxicity tests. However, it is important to highlight that the environmental complexity and interactions between the contaminants and physicochemical variables in rivers were not captured by our study. Additionally, the model organisms most likely belong to a new species that needs to be further described and studied. Many insect species and microbial organisms differ in their interaction with chemical or biological pesticides in aquatic or terrestrial habitats [54,55,56], according to the composition of the toxins and mode of actions. Moreover, future steps could include quantifying pesticide concentrations in the rivers along land use gradients, while simultaneously assessing changes in the structure of aquatic communities and organism stress using biomarkers [57]. Another future research line that deserves attention is the importance of riverine forests as buffers against pesticide pollution in streams surrounded by agricultural fields. Finally, we aim to incorporate higher ecological complexity in future studies, as the ecological effects of pesticides in river ecosystems can be affected by multiple factors (e.g., the geology of the catchment, the interaction with other stressors and the composition of the biological communities, among others).

## Data Availability

Not applicable.
